# Incidence of vertebral fracture in a cohort of Australian women: data from the Geelong Osteoporosis Study

**DOI:** 10.1007/s11657-026-01732-7

**Published:** 2026-07-03

**Authors:** Kara B. Anderson, Amelia G. Morse, Amanda L. Stuart, Julie A. Pasco, Mark A. Kotowicz, Kara L. Holloway-Kew

**Affiliations:** 1 School of Medicine, Deakin Institute for Mental and Physical Health and Clinical Translation, Deakin University, PO Box 183, Geelong, VIC 3220 Australia; 2 Barwon Health, Geelong, Australia; 3 Department of Medicine-Western Health, The University of Melbourne, St Albans, Australia; 4 Department of Epidemiology and Preventive Medicine, Monash University, Melbourne, Australia

**Keywords:** Prevalence, Incidence, Vertebral fracture, Cohort study, Lateral vertebral assessment

## Abstract

***Summary*:**

This study reports the incidence rate of vertebral fractures (6.78 fractures per 1000 person-years) in a representative cohort of Australian women and identifies advanced age, prior low trauma fracture, a history of falls and an increase in bone turnover as potential key predictors.

**Purpose:**

This study aimed to assess prevalence and incidence rates of objectively identified vertebral fracture in a representative cohort of Australian women and explore associated factors.

**Methods:**

Serial lateral vertebral assessments (Lunar Prodigy) were performed in the Geelong Osteoporosis Study (median interval duration 6.86 years; interquartile range: 6.13–7.32). Prevalent and incident vertebral fractures were defined using Genant semi-quantitative approaches, with rates age-standardised to the 2006 Australian population in alignment with the period of data collection. Demographic, clinical and lifestyle factors were collected via a combination of in-person assessment and questionnaires, and logistic regression was used to investigate relationships between these factors and fracture outcomes.

**Results:**

At baseline, 1013 women (ages 20–93 years) were measured and 23 individuals were identified with a prevalent vertebral fracture; 701 women were assessed at follow-up, 19 of whom experienced an incident vertebral fracture. The age-adjusted prevalence at baseline was 2.0% (1.3–2.8), and the age-adjusted incidence rate was 6.78 (4.19–9.37) fractures per 1000 person-years. Prior fracture (OR 6.70, 95% CI 2.55–17.64), lower lumbar spine bone mineral density (OR 0.01, 95% CI 0.00–0.13) and limited physical function (OR 3.80, 95% CI 1.47–9.83) were independently associated with prevalent fracture. In contrast, increased age (OR 1.12, 95% CI 1.06–1.17), prior fracture (OR 3.51, 95% CI 1.15–10.76), a history of falls (OR 3.55, 95% CI 1.29–9.79) and increased markers of bone formation (OR 1.02, 95% CI 1.00–1.04) were associated with incident fracture.

**Conclusions:**

This study reports the incidence rate of vertebral fractures in a representative cohort of Australian women and identifies advanced age, prior low trauma fracture, a history of falls and increased bone turnover as potential key predictors.

**Supplementary information:**

The online version contains supplementary material available at 10.1007/s11657-026-01732-7.

## Introduction

Vertebral fractures are a common consequence of osteoporosis (18 to 26% prevalence depending on geographical region and study methodologies), with data suggesting that rates are higher in younger men compared to similarly aged women but higher in older women [1, 2]. Clinically, however, many vertebral fractures are asymptomatic and may go undetected for considerable amounts of time [3, 4]. Previous literature suggests that vertebral fractures predict future fractures, including those of the hip, where outcomes are poor [5–7], and identifying vertebral fractures soon after occurrence may provide a key opportunity for intervention and prevention [8].

Historically, vertebral fractures have been identified through a range of techniques. Radiological assessment of the vertebral bodies provides detailed information [9], but definitions of vertebral fracture can vary between research studies and may not represent the process undertaken in the clinical environment. A number of proposed quantitative methods such as the Black [10] and Eastell [11] techniques rely on changes in vertebral height compared to adjacent vertebra; however, these processes are time consuming and require significant expertise [12]. The Genant semi-quantitative approach provides an alternative method that uses less precise estimates of percentage height loss to define different grades of deformity or fracture: mild, moderate and severe [13]. The Genant technique has been applied to plain film radiographs as well as lateral images of the spine from dual energy x-ray absorptiometry (DXA) with strong agreement between the two imaging modalities (kappa = 0.84) [14, 15]. DXA approaches thus provide cheaper assessment of morphological vertebral fractures, with significantly less radiation exposure to the patient than plain film x-ray.

The prevalence of vertebral fractures has been established, with rates varying by geographical location, sex and age under investigation [16–20]. Incidence, however, is more difficult to quantify. Some studies measure the incidence of vertebral fracture based on International Classification of Disease (ICD) codes in hospital data and radiology records [21]. For example, previous work by our group suggests the incidence of clinical vertebral fracture to be between 2.1 and 2.2 per 1000 person-years (p/y) in women and 0.7 and 1.3 per 1000 p/y in men [2, 22]. Likewise, the Global Burden of Disease Study reported that age-standardised incident rates for vertebral fracture were 109 per 100000 p/y [18]. This method of fracture ascertainment, however, only captures fractures from presentations of clinical symptoms or incidental findings and likely underestimates the true incidence rate. More comprehensive approaches including longitudinal studies with repeat spine assessment have estimated the incidence of vertebral fracture to vary from 5.2 to 88 fractures per 1000 p/y; however, studies varied in recruitment and fracture ascertainment techniques, which limit their applicability [23–26]. To the authors’ knowledge, there are currently no studies that have reported the incidence of vertebral fracture in the Australian population using semi-quantitative approaches.

Therefore, the aim of this study was to determine the incidence of vertebral fracture in a representative sample of the Australian population of women using comprehensive assessment methods and explore factors associated with fracture incidence.

## Methods

### Study design and participants

**Fig. 1:**
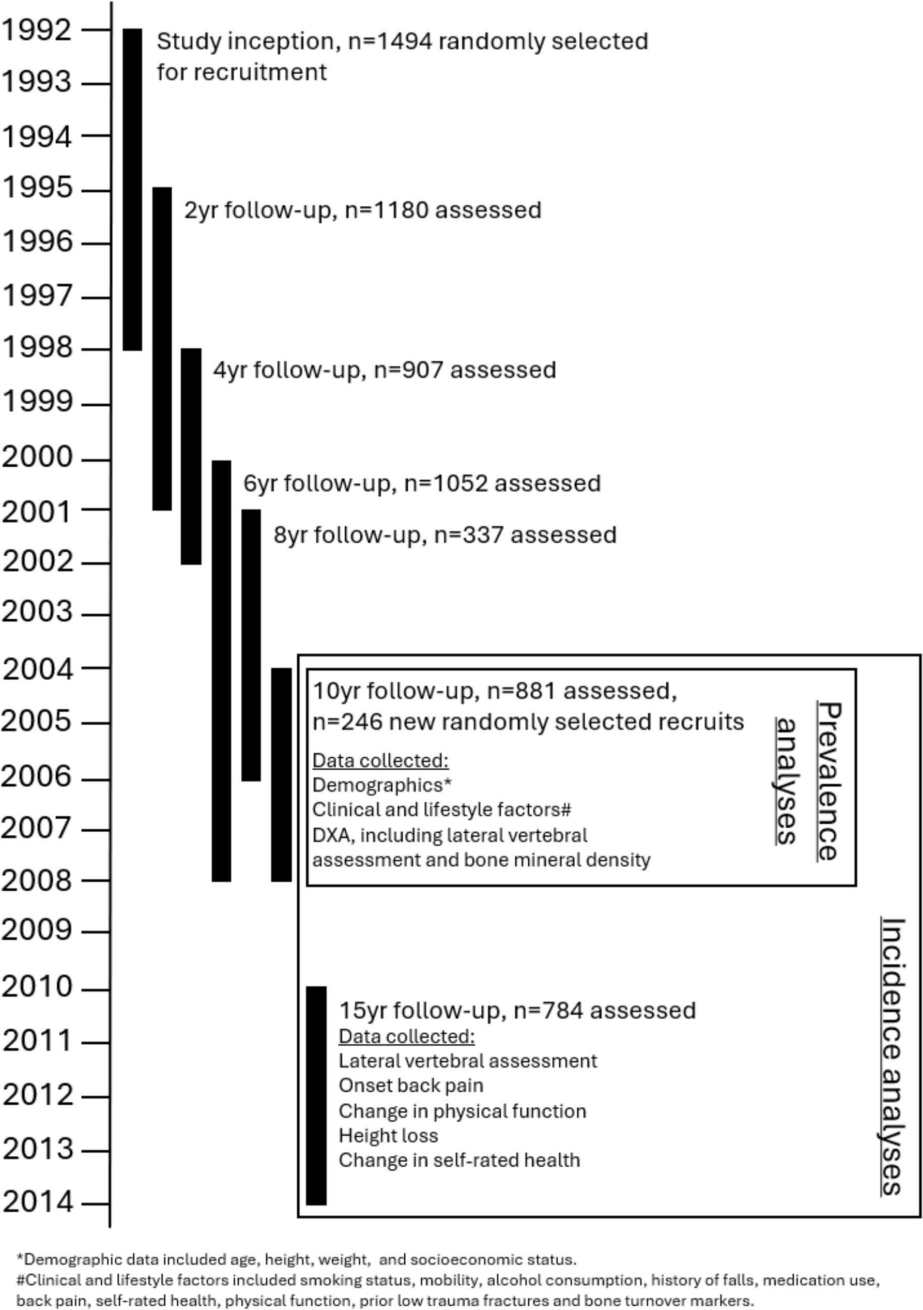
Study design timeline; figure includes date ranges and participant numbers for each of the follow-ups of the Geelong Osteoporosis Study, as well as data collected at each of the time points relevant to the current analyses

**Fig. 2:**
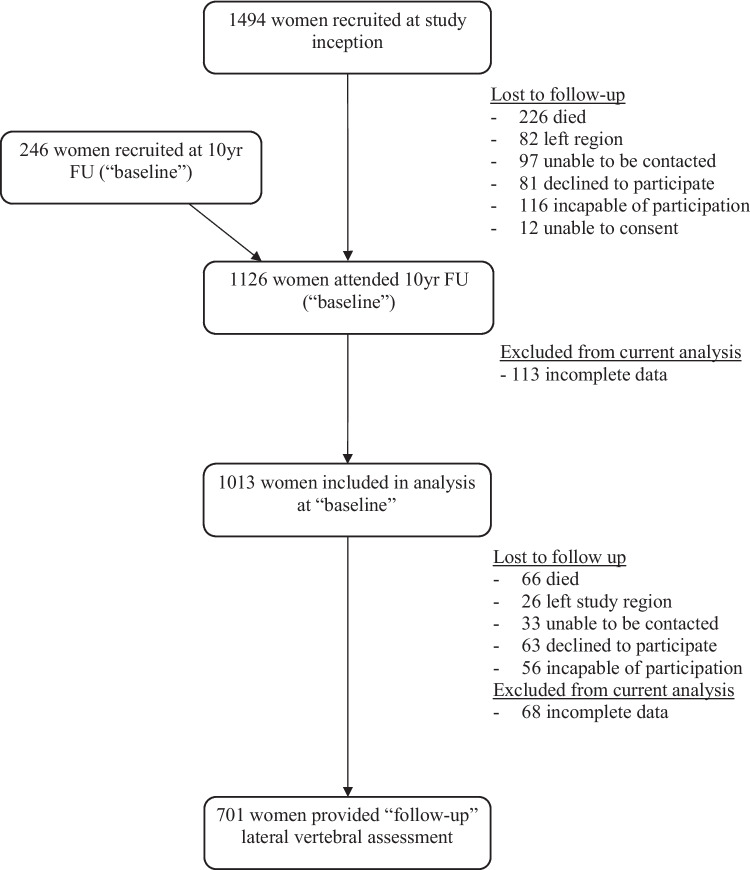
Flow chart of participant inclusion

The Geelong Osteoporosis Study (GOS) is a prospective cohort study in south-eastern Australia that began recruitment of women in 1993 using a random sampling approach [27]. Participants were recruited from electoral rolls incorporating the Barwon Statistical Division, where all citizens aged over 18 years are required to be enrolled, resulting in a comprehensive sampling frame of Australian adults at time of recruitment. Most (~ 99%) of the participants are white and span the entire adult age-range. Participants have been recalled at 2, 4, 6, 8, 10 and 15 year intervals (see Fig. 1) since enrolment in the study, with invitation to return made by mail and subsequent follow-up by phone. Many of the specific reasons for loss to follow-up preclude participation (e.g. death, unable to consent and incapable of participation). Full details of participant inclusion and exclusion are found in Fig. 2. Additionally, a cohort of young women was recruited at the 10-year follow-up using the same recruitment methodology.

The analyses in this paper draw from the 10-year (2004 to 2008) and 15-year follow-up assessment phases (2011 to 2014) and for the purpose of measuring prevalence include all women who provided DXA lateral vertebral assessments at the 10-year visit. For analyses regarding incidence, only those who returned for repeat assessment at the 15-year visit were included. For clarity of understanding in this paper, the 10-year visit will be referred to as “baseline”, and the 15-year visit as “follow-up”.

### Dual x-ray absorptiometry and lateral vertebral assessment

A Lunar Prodigy densitometer (Lunar Prodigy Pro, Madison, WI, USA) was used to perform scans of the lateral spine. A Lunar DPX-L (Madison, WI, USA) was used to perform scans of the anteroposterior lumbar spine and hip. Lateral spine scans were analysed for vertebral fracture assessment at the lower thoracic and lumbar spine regions according to the software protocol utilising the Genant semi-quantitative approach at both time points (baseline and follow-up). This technique uses reductions in vertebral height and area to define fractures. A reduction of 25–40% in anterior, middle and/or posterior height and a reduction in area of 20–40% defined a moderate fracture, and a reduction of 40% or more defined a severe fracture. Mild vertebral fractures were not captured via this technique due to previously published poor agreement between radiography and DXA assessment for these fractures [14]. Incident vertebral fracture was defined as a fracture not present at baseline that was later identified at follow-up.

### Anthropometry and other confounders

Anthropometry and potential confounders were measured and reported at baseline (10-year follow-up), with some key variables re-assessed over time, as detailed below. Height was measured without shoes using a wall-mounted Harpenden stadiometer to the nearest 0.1 cm; weight was measured in a hospital gown or minimal clothing on electronic scales to the nearest 0.1 kg. Body mass index (BMI) was calculated as kg/cm^2^. Height loss was determined where participants’ height at follow-up was measured as more than 2 cm below their baseline height. Smoking status was defined as either current smoker or non-smoker, based upon self-report of smoking of cigarettes or other tobacco products at the time of assessment. Mobility was measured using a seven-point mobility scale, dichotomised into “low mobility” (sedentary, limited, inactive, bed/chair ridden, bedfast) and “high mobility” (active, very active). Alcohol consumption was calculated using the Cancer Council Food Frequency Questionnaire (FFQ) [28]. Low alcohol consumption was defined as 0–2 standard drinks (20 g or less of alcohol) per day, in accordance with the Australian National Health and Medical Research Council recommendations [29]. A history of falls in the year prior to participant appointment was self-reported, as well as the use of antifracture medication and medications that affect bone and calcium metabolism. Participants were asked to bring in any current medications at the time of their appointment for verification by research staff. Back pain “during the past week” was reported at both visits on a 100-mm visual analogue scale and converted to a binary variable where “pain” was a mark on the scale greater than or equal to 1 mm [30]. Participants were considered to have “interval onset back pain” if they reported pain at follow-up but not at baseline. Participants self-reported their health on a 5-point scale: “excellent”, “very good”, “good”, “fair” or “poor”, with decline in self-rated health being defined as a decrease from one category to a lower category over the follow-up period. Limited physical function was defined from a two-part question where participants were asked if their health limited them in undertaking (a) moderate activities such as moving a table, pushing a vacuum cleaner, bowling or playing golf, or (b) climbing several flights of stairs. Participants who answered “yes” to either question were defined as having limited physical function, and a decline in physical function was defined by a change in status from no limitation to being limited over the follow-up period. Socioeconomic status was determined based on reported address at time of visit using the Australian Bureau of Statistics Index of Relative Socio-economic Advantage and Disadvantage and aggregated into quintiles. Prior low trauma fracture in adulthood (after age 20 years) was initially self-reported and then followed up by radiological report where possible. Serum C-terminal cross-linked telopeptide (CTX; ng/L) and procollagen type 1 N-terminal propeptide (P1NP; µg/L) bone turnover markers were analysed using the automated Roche Modular Analytics E170 analyser (Roche Diagnostics, Mannheim, Germany).

### Statistical analysis

Summary statistics were reported using mean and standard deviation (SD) or median and interquartile range (IQR) as appropriate. Inter-group differences for participants with and without a vertebral fracture were assessed using independent *t*-tests and Mann-Whitney *U*-tests. Categorical data were described as *n* (%), and between group differences were assessed using chi-square statistics. Prevalence rate was defined as the percentage of participants with a vertebral fracture, and incidence rate was calculated as the number of participants who sustained a fracture over the total number of p/y under observation. Both prevalence and incidence were age-standardised to the Australian population using 2006 census data in alignment with the period of baseline data collection. Multivariate logistic regression modelling was further used to explore the combined effects of potential factors associated with prevalent and incident fracture. Variables identified as significantly different between fracture and fracture-free groups were tested in models and retained if significant (*p* < 0.05) through a backward stepwise approach. Odds ratios (OR) and 95% confidence intervals (95%CI) are reported. In addition, sensitivity analyses reporting summary statistics, rates and logistic regression modelling for participants aged 50 years and over were also completed. All analyses undertaken in this study were conducted using StataSE 18 (StataCorp. 2018. *Stata Statistical Software: Release 18.* College Station, TX: StataCorp LLC).

## Results

### Prevalence of vertebral fractures

At baseline, 1013 women underwent lateral vertebral assessment. Of these, 23 women were identified as having at least one prevalent fracture, with five individuals having two or more fractures for a total of 33 fractures. Of these, 20 were classified as “moderate” and 13 were classified as “severe”. The age-adjusted prevalence was 2.0% (95% CI 1.3–2.8%).

Descriptive characteristics of these participants are listed in Table 1, by prevalent fracture status. Women identified with a prevalent fracture were older and shorter than their fracture-free counterparts. They also had lower BMD at both the hip and spine, and reduced bone formation as measured by lower serum P1NP. These women were more likely to report lower mobility and to have a history of low trauma fracture. They were more likely to report limits in their physical function, but not more likely to report back pain, and were more likely to use an antiresorptive medication. Differences in self-rated health were also observed between groups. With regard to medication usage, of the 21 participants using a bisphosphonate, the median duration of use was 3 years (ranging from less than 1 year to approximately 10 years). Of 14 participants using a glucocorticoid, the median duration of use was 2 years (ranging from less than 1 year to 19 years).

**Table 1:** Descriptive characteristics of all participants and participants aged 50 years and over according to prevalent vertebral fracture status at baseline. Continuous variables presented as mean ± SD or median (IQR) as appropriate and categorical variables as *n* (%). *p*-values refer to inter-group differences

	Full cohort			Ages 50 yr and older		
	Prevalent vertebral fracture (*n* = 23)	No prevalent vertebral fracture (*n* = 990)	*p****-***value	Prevalent vertebral fracture (*n* = 18)	No prevalent vertebral fracture (*n* = 494)	*p****-***value
Age (yr)	70.7 (54.2–78.5)	49.9 (33.7–64.2)	<0.001	75.1 (67.0–79.9)	64.2 (57.4–73.6)	0.002
Height (cm)	156.9 ± 6.0	162.5 ± 6.7	<0.001	156.1 ± 5.2	161.0 ± 6.5	0.002
Weight (kg)	63.5 (56.8–78.9)	69.4 (61.5–80.6)	0.219	64.0 (58.3–78.9)	70.1 (61.8–81.1)	0.407
Body mass index (kg/m^2^)	27.0 (23.2–31.6)	26.2 (23.3–30.6)	0.892	27.7 (24.1–31.6)	26.9 (23.9–31.1)	0.998
Lumbar spine BMD (g/cm^2^)	1.014 ± 0.145	1.216 ± 0.185	<0.001	0.995 ± 0.136	1.174 ± 0.195	<0.001
Femoral neck BMD (g/cm^2^)	0.779 ± 0.132	0.947 ± 0.163	<0.001	0.729 ± 0.081	0.896 ± 0.159	<0.001
CTX (ng/L)	369 (206–493)	356 (266–474)	0.200	401 (206–521)	381 (285–491)	0.090
P1NP (µg/L)	40 (22–77)	50 (38–64)	0.019	51 (22–80)	52 (39–66)	0.078
Fall in the past year	9 (39.1)	250 (25.3)	0.134	9 (50.0)	143 (29.1)	0.057
Low mobility	14 (60.9)	191 (19.3)	<0.001	11 (61.1)	120 (24.3)	<0.001
Alcohol consumption*	5 (21.7)	277 (28.8)	0.461	4 (22.2)	130 (27.1)	0.648
Prior adulthood low trauma fracture	16 (69.6)	137 (13.8)	<0.001	16 (88.9)	90 (18.2)	<0.001
Back pain	10 (43.5)	393 (40.3)	0.756	9 (50.0)	200 (41.3)	0.463
Self-rated health			0.002			0.001
Excellent	3 (13.0)	158 (16.0)		2 (11.1)	71 (14.5)	
Very good	9 (39.1)	427 (43.3)		8 (44.4)	193 (39.3)	
Good	6 (26.1)	295 (29.9)		4 (22.2)	168 (34.2)	
Fair	2 (8.7)	92 (9.3)		1 (5.6)	50 (10.2)	
Poor	3 (13.0)	15 (1.5)		3 (16.7)	9 (1.8)	
Limited physical function	16 (69.6)	274 (27.7)	<0.001	14 (77.8)	171 (34.8)	<0.001
Smoker	2 (8.7)	147 (14.9)	0.409	1 (5.6)	54 (11.0)	0.468
Antiresorptive use	5 (21.7)	16 (1.6)	<0.001	5 (27.8)	12 (2.4)	<0.001
Glucocorticoid use	1 (4.4)	13 (1.3)	0.218	1 (5.6)	9 (1.8)	0.262
Socioeconomic status			0.980			0.996
Quintile 1 (low)	3 (13.0)	159 (16.1)		3 (16.7)	87 (17.6)	
Quintile 2	5 (21.7)	205 (20.7)		3 (16.7)	92 (18.6)	
Quintile 3	6 (26.1)	223 (22.5)		4 (22.2)	120 (24.3)	
Quintile 4	5 (21.7)	197 (19.9)		4 (22.2)	98 (19.8)	
Quintile 5	4 (17.4)	206 (20.8)		4 (22.2)	97 (19.6)	

*CTX*: C-terminal cross-linked telopeptide, *P1NP*: procollagen type 1 N-terminal propeptide

^*^Alcohol consumption > 2 standard drinks per day

Logistic regression modelling showed a number of independent associations with prevalent fracture, including lumbar spine BMD (OR 0.01; 95% CI 0.00–0.13), prior low trauma fracture (OR 6.70; 95% CI 2.55–17.64) and limited physical function (OR 3.80, 95% CI 0.06–26.07).

When considering only those aged 50 years and over, associations were largely sustained, although there was no significant difference in either bone turnover marker in this age group (see Table 1). Age-standardised prevalence when restricted to this older cohort of women aged over 50 years was 3.3% (95% CI 1.9–4.8%). Logistic regression modelling showed similar associations with slightly higher effect sizes and considerably larger confidence intervals (data not shown).

### Incidence of vertebral fractures

At follow-up, 701 women returned for lateral vertebral assessment. Of the 312 participants who did not return for follow-up, 66 had died during the follow-up period (cancer *n* = 22, conditions of the circulatory system *n* = 20, diseases of the digestive system *n* = 4, dementia *n* = 4, diseases of the respiratory system *n* = 3, Parkinson’s disease *n* = 2, other [incl. accident and injury, musculoskeletal system disease and blood disease] *n* = 11). Women lost to follow-up were older, slightly shorter and lighter and had lower BMD at both the hip and lumbar spine (see Supplemental Table 1). They also had higher bone turnover compared to peers who returned for follow-up and were more likely to have lower mobility, a prior low trauma fracture, poorer self-rated health and poorer physical function. They were also more likely to use an antiresorptive medication.

Incident vertebral fractures were identified in 19 participants, for a total of 24 fractures. Of these, 15 were considered “moderate” and 9 were considered “severe”. The median follow-up time of participants was 6.86 (IQR 6.13–7.32) years, with a total of 4703.38 p/y of observation. The age-standardised incidence rate of fracture was 6.78 (95% CI 4.19–9.37) per 1000 p/y.

Descriptive characteristics of participants who returned at follow-up are outlined in Table 2, according to the presence or absence of incident vertebral fracture. In brief, individuals who experienced an incident fracture were older and shorter than participants who did not. Participants with fracture were more likely to be fallers, smokers, have lower mobility and an existing vertebral fracture or prior adulthood low trauma fracture at their baseline assessment. They also had lower femoral neck BMD, but differences in lumbar spine BMD did not reach significance. Incident vertebral fracture was not associated with interval onset back pain or height loss but was associated with limited physical function and decline in physical function over the follow-up period.

**Table 2:** Descriptive characteristics of all participants and participants aged 50 years and over according to incident vertebral fracture status at follow-up. Continuous variables presented as mean ± SD or median (IQR) as appropriate and categorical variables as *n* (%). *p*-values refer to inter-group differences

	Full cohort			Ages 50 yr and over		
	Incident vertebral fracture (*n* = 19)	No incident vertebral fracture (*n* = 682)	*p****-***value	Incident vertebral fracture (*n* = 16)	No incident vertebral fracture (*n* = 324)	*p****-***value
Age (yr)	70.1 (62.9–73.8)	48.7 (36.7–60.2)	< 0.001	71.2 (66.4–74.8)	60.9 (55.4–67.3)	< 0.001
Height (cm)	158.1 ± 5.5	163.0 ± 6.4	0.001	156.9 ± 4.9	161.3 ± 6.3	0.006
Weight (kg)	72.2 (64.4–80.6)	69.4 (61.8–80.2)	0.666	70.6 (64.6–80.1)	71.1 (62.5–81.2)	0.718
Body mass index (kg/m^2^)	30.0 (26.7–31.9)	26.1 (23.3–30.4)	0.030	30.0 (27.0–31.8)	26.9 (24.3–31.2)	0.198
Lumbar spine BMD (g/cm^2^)	1.148 ± 0.188	1.225 ± 0.178	0.065	1.115 ± 0.151	1.164 ± 0.188	0.307
Femoral neck BMD (g/cm^2^)	0.845 ± 0.142	0.957 ± 0.151	0.001	0.811 ± 0.123	0.888 ± 0.141	0.032
CTX (ng/L)	401 (332–508)	342 (252–458)	0.060	411 (373–513)	381 (278–493)	0.213
P1NP (µg/L)	62 (52–69)	48 (37–62)	0.031	62 (52–74)	50 (39–65)	0.066
Fall in the past year	10 (52.6)	165 (24.5)	0.005	10 (62.5)	90 (28.0)	0.003
Low mobility	8 (42.1)	92 (13.5)	< 0.001	7 (43.8)	52 (16.1)	0.004
Alcohol consumption*	3 (15.8)	201 (30.0)	0.182	3 (18.8)	101 (31.4)	0.286
Prior adulthood low trauma fracture	6 (31.6)	102 (15.0)	0.048	5 (31.3)	54 (16.7)	0.133
Existing vertebral fracture	2 (10.5)	11 (1.6)	0.005	1 (6.3)	9 (2.8)	0.422
Back pain at follow-up	14 (73.7)	450 (67.3)	0.556	12 (75.0)	217 (68.9)	0.606
Interval onset back pain	9 (47.4)	215 (32.4)	0.172	7 (43.8)	102 (32.8)	0.365
Self-rated health			0.006			0.366
Excellent	1 (5.6)	116 (17.1)		0	36 (11.2)	
Very good	9 (50.0)	277 (40.9)		8 (53.3)	118 (36.5)	
Good	7 (38.9)	230 (34.0)		7 (46.7)	137 (42.4)	
Fair	0	52 (7.7)		0	31 (9.6)	
Poor	1 (5.6)	2 (0.3)		0	1 (0.3)	
Decline in self-rated health	6 (33.3)	168 (24.9)	0.415	5 (33.3)	94 (29.2)	0.731
Limited physical function	14 (73.7)	269 (39.7)	0.003	12 (75.0)	191 (59.0)	0.201
Decline in physical function	10 (52.6)	153 (22.6)	0.002	9 (56.3)	105 (32.4)	0.049
Height loss > 2 cm	2 (10.5)	78 (11.4)	0.902	2 (12.5)	63 (19.4)	0.490
Smoker	8 (42.1)	87 (12.8)	< 0.001	7 (43.8)	50 (15.4)	0.003
Antiresorptive use	0	8 (1.2)	-	0	7 (2.2)	0.552
Glucocorticoid use	0	8 (1.2)	-	0	6 (1.9)	0.583
Socioeconomic status			0.054			0.096
Quintile 1 (referent)	7 (36.8)	102 (14.9)		6 (37.5)	50 (15.4)	
Quintile 2	4 (21.1)	135 (19.8)		4 (25.0)	66 (20.4)	
Quintile 3	5 (26.3)	148 (21.7)		4 (25.0)	74 (22.8)	
Quintile 4	1 (5.3)	142 (20.8)		1 (6.3)	62 (19.1)	
Quintile 5	2 (10.5)	155 (22.7)		1 (6.3)	72 (22.2)	

*CTX* C-terminal cross-linked telopeptide, *P1NP* procollagen type 1 N-terminal propeptide

^*^Alcohol consumption > 2 standard drinks per day

Logistic regression modelling found an independent contribution of age (OR 1.12, 95% CI 1.06–1.17), P1NP (OR 1.02, 95% CI 1.01–1.04), falls in the past year (OR 1.29, 95% CI 1.29–9.79) and prior fracture (OR 3.51, 95% CI 1.15–10.76) to incident fracture.

When only those individuals aged 50 years and over were considered, associations were similar (see Table 2). However, the relationship between vertebral fracture and BMI, bone turnover markers, prior fracture, self-rated health and baseline physical function was attenuated. As in the full age range, there was no association with back pain in this age-group. The age-standardised incidence rate was 13.66 (95% CI 8.44–18.89) per 1000 p/y.

Logistic regression modelling for older women showed similar associations; however, prior fracture was no longer a significant independent contributor (data not shown).

## Discussion

The current study observed an incidence of vertebral fractures at a rate of 6.78 (4.19–9.37) per 1000 p/y across the entire population aged 20 years and older. In older women, the rate was higher (13.66; 95% CI 8.44–18.89 per 1000 p/y), which aligns with published data. For example, the CaMos study, a population-based, randomly selected study of Canadian women and men aged 50 years and over, reported incidence rates to vary from 6.3 to 10.2 fractures per 1000 p/y, depending on the methodological approach used to determine fracture [23]. The CaMos reported no difference in rates between men and women but did find that rate increased with increasing age, much as in the current study.

In the Rotterdam study, which invited the entire population of Oomoord, Rotterdam in the Netherlands aged 55 years and over, the incident fracture rate was 10.9 per 1000 p/y, but higher in women than men (14.7 vs 5.9 per 1000 p/y) [25]. They reported that individuals who experienced an incident fracture were more likely to use a walking aid (as a stand-in for poor mobility) and to be a current smoker compared to those who did not sustain a fracture during the average 6.3 years of follow-up [25]. These data align with the demographic data presented in the current study.

Conversely, in Thailand, a prospective cohort study of residents in Romklao, Bangkok, aged 50 years and over reported that overall incidence of vertebral fracture was 39.7 per 1000 p/y [24], and the rate was lower in women than in men (32.1 and 54.5 per 1000 p/y, respectively). This is higher than in the European and North American studies, and factors associated with incident fracture included age, sex and the presence of prior vertebral fractures [24].

Finally, in a Japanese study, rates were substantially higher, ranging from 5.2 to 88 vertebral fractures per 1000 p/y, depending on age, sex and prevalent fracture [26]. These participants ranged from 47 to 92 years, similar to ours and other studies, yet it should be noted that the cohort study, the Adult Health Study, was established to study the effects of radiation exposure on atomic bomb survivors and included about 15,000 survivors and 5000 controls, and this sampling frame may have biased the results [26].

With regard to factors associated with prevalent and incident fracture, we identified a number of variables that may be of potential interest. Many of these are evident in the existing literature such as age, BMI, BMD, bone turnover, falls and prior fracture [31–33]. Of note, we also identified differences in self-rated health and physical function (both at baseline and decline over time) between those with and without an incident fracture, but no difference in reported back pain. This aligns with previous data in men that found no association between back pain and incident fracture [34].

This study has significant strengths, including the representative nature of the cohort. The participants of the study were not selected on the basis of disease and were randomly selected from a comprehensive sampling frame of the population within a clearly defined geographical area, suggesting that the incidence rate is a reliable estimate for the underlying population. We acknowledge that this study assessed women only, most of whom were white; therefore, the results may not be applicable to other populations including men, non-white women or individuals in other geographical areas. The semi-quantitative approach to fracture assessment ensured that all participants were assessed in a consistent manner. However, there is likely to be some healthy participant bias, both at recruitment and retention during follow-up. As shown in Supplement Table 1, participants who were lost to follow-up were more likely to have factors that may have contributed to overall poorer health and risk of fracture specifically, and this may have influenced results.

The use of vertebral fracture assessment via DXA in this study may have underestimated the true rate of fracture, as the technique is less sensitive than expert assessment of plain radiography films [14]. However, it is inappropriate to routinely capture plain film radiography for population-based participants due to the significant exposure to radiation and higher cost. The use of standardised vertebral fracture assessment software as part of the Lunar Prodigy package ensures that the Genant semi-quantitative approach is consistently applied to all participant scans.

Regarding statistical analysis, we acknowledge the small absolute number of fractures observed, which may have impacted our findings, particularly in relation to tests of difference between groups and regression modelling, where large confidence intervals were observed. We present these findings with this consideration in mind.

This work builds upon our previous work investigating prevalence of vertebral fractures in a similar cohort of men [34]. Future work will explore incidence rates in men, and since sex-based differences in the incidence of vertebral fractures are likely, investigating this avenue will provide a more well-rounded picture as to the state of vertebral fractures in the population. Further, a larger sample size and longer follow-up period would allow for the investigation of multiple vertebral fractures over time and the effect of existing fractures on future incidence.

## Conclusion

In an Australian population of women, using DXA vertebral fracture assessment by the Genant semi-quantitative method, vertebral fractures occurred at a rate of 6.78 fractures per 1000 p/y for ages 20+ and 13.66 for older women aged 50+, with age, history of falling, reduced mobility and current use of tobacco products associated with increased risk of fracture.

## Supplementary information

Below is the link to the electronic supplementary material.

ESM 1 (DOCX 25.2 KB)

## Data Availability

Data may be available from the research team upon reasonable request.
