# Supplementary material for: Incidence of vertebral fracture in a cohort of Australian women: data from the Geelong Osteoporosis Study
Source: Arch Osteoporos. 2026 Jul 3;21(1):99. doi: 10.1007/s11657-026-01732-7 (PMC13331909; doi:10.1007/s11657-026-01732-7)
Supplement: Supplementary file 1 — (DOCX 25.2 KB) [file 11657_2026_1732_MOESM1_ESM.docx]

**Supplemental Table 1.** Descriptive characteristics of all participants according to attendance at follow-up visit. Continuous variables presented as mean±SD or median (IQR) as appropriate and categorical variables as n (%). *P*-values refer to inter-group differences.

|  | **All (n=1,013)** | **Did not attend follow-up (n=312)** | **Attended follow-up (n=701)** | ***p-*value** |
| --- | --- | --- | --- | --- |
| **Age (yr)** | 50.3 (33.8-64.7) | 54.9 (28.8-72.6) | 49.8 (37.2-61.8) | <0.001 |
| **Height (cm)** | 162.4±6.8 | 161.4±7.4 | 162.9±6.4 | 0.002 |
| **Weight (kg)** | 69.4 (61.4-80.6) | 68.9 (59.8-82.1) | 69.4 (61.8-80.2) | 0.037 |
| **Body mass index (kg/m^2^)** | 26.2 (23.3-30.6) | 26.6 (22.9-31.2) | 26.2 (23.4-30.5) | 0.220 |
| **Lumbar spine BMD (g/cm^2^)** | 1.211±0.186 | 1.185±0.201 | 1.223±0.179 | 0.004 |
| **Femoral neck BMD (g/cm^2^)** | 0.943±0.164 | 0.918±0.186 | 0.954±0.152 | 0.001 |
| **CTX (ng/L)** | 356 (262-474) | 387 (278-508) | 345 (253-462) | 0.010 |
| **P1NP (µg/L)** | 50 (38-64) | 54 (38-69) | 49 (38-63) | 0.006 |
| **Fall in the past year** | 259 (25.6) | 84 (27.1) | 175 (25.0) | 0.482 |
| **Low mobility** | 205 (20.3) | 105 (33.7) | 100 (14.3) | <0.001 |
| **Alcohol consumption*** | 282 (28.6) | 78 (26.4) | 204 (29.6) | 0.306 |
| **Prior adulthood low trauma fracture** | 153 (15.1) | 66 (21.2) | 87 (12.4) | <0.001 |
| **Back pain** | 403 (40.3) | 130 (42.8) | 273 (39.3) | 0.302 |
| **Self-rated health** |  |  |  | 0.001 |
| **Excellent** | 161 (15.9) | 46 (14.8) | 115 (16.5) |  |
| **Very Good** | 436 (43.2) | 111 (35.7) | 325 (46.5) |  |
| **Good** | 301 (29.8) | 105 (33.8) | 196 (28.0) |  |
| **Fair** | 94 (9.3) | 40 (12.9) | 54 (7.7) |  |
| **Poor** | 18 (1.8) | 9 (2.9) | 9 (1.3) |  |
| **Limited physical function** | 290 (28.7) | 119 (38.3) | 171 (24.4) | <0.001 |
| **Smoker** | 149 (14.7) | 54 (17.3) | 95 (13.6) | 0.121 |
| **Antiresorptive use** | 21 (2.1) | 13 (4.2) | 8 (1.1) | 0.002 |
| **Glucocorticoid use** | 14 (1.4) | 5 (1.6) | 9 (1.3) | 0.690 |
| **Socioeconomic status** |  |  |  | 0.280 |
| **Quintile 1 (low)** | 162 (16.0) | 53 (17.0) | 109 (15.6) |  |
| **Quintile 2** | 210 (20.7) | 71 (22.8) | 139 (19.8) |  |
| **Quintile 3** | 229 (22.6) | 76 (24.4) | 153 (21.8) |  |
| **Quintile 4** | 202 (19.9) | 59 (18.9) | 143 (20.4) |  |
| **Quintile 5** | 210 (20.7) | 53 (17.0) | 157 (22.4) |  |
| *Alcohol consumption >2 standard drinks per day.  CTX=C-terminal cross-linked telopeptide; P1NP=procollagen type 1 N-terminal propeptide | | | |  |
